# MUC1 glycopeptide epitopes predicted by computational glycomics

**DOI:** 10.3892/ijo.2012.1645

**Published:** 2012-09-27

**Authors:** WEI SONG, ELIZABETH S. DELYRIA, JIEQING CHEN, WEI HUANG, JUN SOO LEE, ELIZABETH A. MITTENDORF, NUHAD IBRAHIM, LASZLO G. RADVANYI, YUNSEN LI, HONGZHOU LU, HUAXI XU, YINQIANG SHI, LAI-XI WANG, JEREMY A. ROSS, SILAS P. RODRIGUES, IGOR C. ALMEIDA, XIFENG YANG, JIN QU, NATHANIEL S. SCHOCKER, KATJA MICHAEL, DAPENG ZHOU

**Affiliations:** 1 Departments of Melanoma Medical Oncology; 2 Surgical Oncology and; 3 Breast Medical Oncology, The University of Texas M.D. Anderson Cancer Center, Houston, TX;; 4 Institute of Human Virology and Department of Biochemistry and Molecular Biology, University of Maryland School of Medicine, Baltimore, MA;; 5 Institutes of Biology and Medical Sciences, Soochow University, Suzhou;; 6 Shanghai Public Health Clinical Center, Shanghai;; 7 School of Medical Science and Laboratory Medicine, Jiangsu University, Zhenjiang;; 8 Department of Gastric Cancer and Soft Tissue Sarcomas, Fudan University Shanghai Cancer Center, Shanghai 200032, P.R. China;; 9 Department of Biological Sciences, Border Biomedical Research Center, University of Texas at El Paso, El Paso, TX;; 10 BioLegend Inc., San Diego, CA, USA;; 11 College of Bioscience and Biotechnology, Yangzhou University, Yangzhou, P.R. China;; 12 Department of Chemistry, University of Texas at El Paso, El Paso, TX, USA

**Keywords:** computational glycomics, MUC1, glycopeptide, monoclonal antibodies, cancer, immunopathogenesis, immunotherapy

## Abstract

Bioinformatic tools and databases for glycobiology and glycomics research are playing increasingly important roles in functional studies. However, to verify hypotheses generated by computational glycomics with empirical functional assays is only an emerging field. In this study, we predicted glycan epitopes expressed by a cancer-derived mucin, MUC1, by computational glycomics. MUC1 is expressed by tumor cells with a deficiency in glycosylation. Although numerous diagnostic reagents and cancer vaccines have been designed based on abnormally glycosylated MUC1 sequences, the glycan and peptide sequences responsible for immune responses *in vivo* are poorly understood. The immunogenicity of synthetic MUC1 glycopeptides bearing Tn or sialyl-Tn antigens have been studied in mouse models, while authentic glyco-epitopes expressed by tumor cells remain unclear. To examine the immunogenicity of authentic cancer derived MUC1 glyco-epitopes, we expressed membrane bound forms of MUC1 tandem repeats in Jurkat, a mutant cancer cell line deficient of mucin-type core-1 β1–3 galactosyltransferase activity, and immunized mice with cancer cells expressing authentic MUC1 glyco-epitopes. Antibody responses to individual glyco-epitopes were determined by chemically synthesized candidate MUC1 glycopeptides predicted through computational glycomics. Monoclonal antibodies can be generated toward chemically synthesized glycopeptide sequences. With RPAPGS(Tn)TAPPAHG as an example, a monoclonal antibody 16A, showed 25-fold higher binding to glycosylated peptide (EC_50_=9.278±1.059 ng/ml) compared to its non-glycosylated form (EC50=247.3±16.29 ng/ml) as measured by ELISA experiments with plate-bound peptides. A library of monoclonal antibodies toward authentic MUC1 glycopeptide epitopes may be a valuable tool for studying glycan and peptide sequences in cancer, as well as reagents for diagnosis and therapy.

## Introduction

A characteristic feature of epithelial cancer is aberrant glycosylation of glycoproteins. The Tn antigen, an O-linked N-acetylgalactosamine (GalNAc) epitope, which should be covered by other distal sugars in normal cells, is a well characterized glyco-epitope expressed by breast, colorectal and ovarian cancer cells (1–8).

MUC1 is a 500–1000-kDa transmembrane glycoprotein expressed by normal and cancer cells. In healthy human epithelial cells MUC1 is expressed on the apical surface of the cell, i.e., the side of the cell membrane that faces the tubular interior of a vessel. As a mucin, its functions are to lubricate, to keep the cell hydrated, and to protect from pathogen invasion. The extracellular domain of MUC1 contains a variable number (25–125) of tandem repeats of 20 amino acids in length (1–8), each of which has five potential O-glycosylation sites: (-His-Gly-Val-**Thr-Ser**-Ala-Pro-Asp-**Thr**-Arg-Pro-Ala-Pro-Gly-**Ser-Thr**-Ala-Pro-Pro-Ala-)_n_. In healthy cells, MUC1 is heavily glycosylated, and the O-glycans are mostly of the core 2 type, which is a trisaccharide. It may be elongated by several LacNAc units, whereas fucose and/or sialic acid are terminal sugars of the completed oligosaccharide. Unlike normal cells, most carcinomas overexpress MUC1, and MUC1 is distributed over the entire cell surface. The glycans are ‘abnormal’ due to incomplete glycosylation and premature sialylation. Two common tumor associated antigens found in carcinomas are the Tn (αGalNAc, 2), and the STn (αNeuAc-2,6-αGalNAc). It is believed that these glycans are truncated because there are different expression levels of glycosyl transferases in carcinomas when compared to healthy cells, which may be caused by mutation or inactivation of glycosyltransfeases, or by lack of functional chaperone proteins for glycosyltransferases. The mutation of COSMC, an X chromosome located gene encoding a chaperon protein required by core-1 β1,3-galactosyltransferase, is believed to be associated with expression of the Tn antigen (9). Due to the overexpression of MUC1 by almost all epithelial carcinomas, glycopeptide partial sequences with abnormal O-glycans contained in the MUC1’s tandem repeats are ideal potential antigens and biomarkers that could be detected by monoclonal antibodies.

A fundamental problem is that the exact epitopes at the molecular level remain unknown. If and how epitope expression changes over time as the cancer progresses is also poorly understood (10). One could envision isolating MUC1 glycopeptides directly from cancer cells, and analyzing them by mass spectrometry. However, a major obstacle is the resistance of MUC1 toward proteolytic digestion. Two methods that have been used for the discovery or detection of MUC1 epitopes both take advantage of glycostructure recognition by antibodies. The first method utilizes synthetic glycopeptides believed to exist on cancer MUC1, conjugation of these glycopeptides to create an immunogen, immunization of mice (11), collection of anti-sera, and analysis of the binding of antibodies to MUC1 expressed on cancer cells by flow cytometry (FACS) analysis (12). The advantage of this method is that polyclonal or monoclonal antibodies can be obtained that recognize an epitope of known chemical structure, and using these antibodies, cancer tissue can be analyzed for the presence of a particular epitope. However, the obtained antibodies can only detect the epitope for which they were elicited, and the presence of other epitopes may remain undetected. The second approach utilizes modern glyco-microarrays (13), which are utilized for the screening of patient serum for the presence of auto-antibodies that recognize compounds in the microarray. While this approach is capable of detecting multiple epitopes given that the patient has produced auto-antibodies, large micro-arrays are required, and not every patient produces sufficient levels of auto-antibodies against MUC1. Thus, cancer may not be detected in all patients due to an insufficient diversity of the microarray, or due to no (or weak) immune responses to the cancer. While large glyco-microarrays have been printed, including glycopeptides of MUC1, undoubtedly even more diverse glyco-microarrays are needed for the discovery of new biomarkers using the screening methodologies already in place. There are currently no tools available that allow for a comprehensive screening of cancer tissue for the presence of a large number of specific biomarkers.

**Figure 1:**
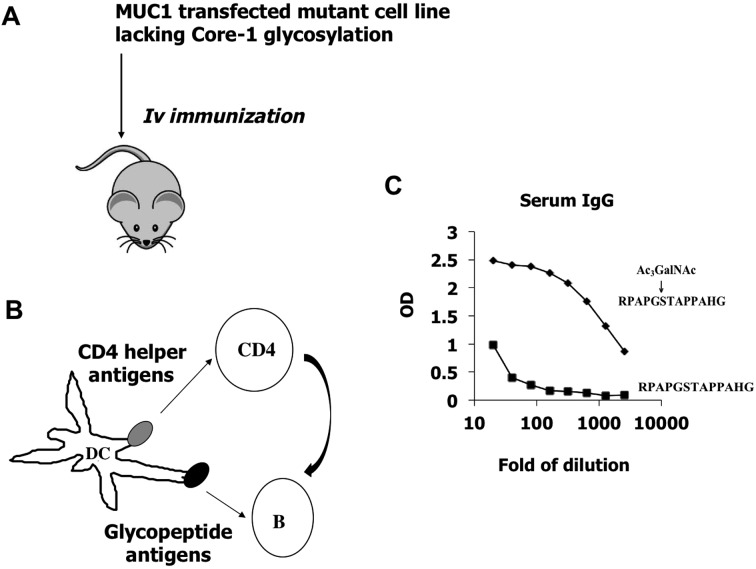
Generation of monoclonal antibodies by immunizing mice with xenogenic tumor cell lines lacking core-1 β3-galactosyltransferase activity. (A) C57B6 strain of mice were intravenously immunized by Jurkat cell line transfected by MUC1 gene; (B) MUC1 epitopes expressed on tumor cell surface stimulate B cells to produce antibodies. Tumor cell antigens provide CD4 T cell help to B cells. (C) Antibody responses toward glycopeptide can be detected by ELISA experiments. Monoclonal antibodies can be generated by specific glycopeptides.

In this study, we generated glycopeptide-specific antibodies which recognize authentic glycoforms expressed by tumor cells (Fig. 1). The method will allow us to generate a library of monoclonal antibodies that recognize all existing forms of glycopeptides abnormally expressed by tumor cells deficient in O-glycosylation, which may lead to feasible methods to study the O-linked glycoproteome in cancer cells.

## Materials and methods

### Predicting glycopeptide sequences by computational glycomics

**Figure 2:**
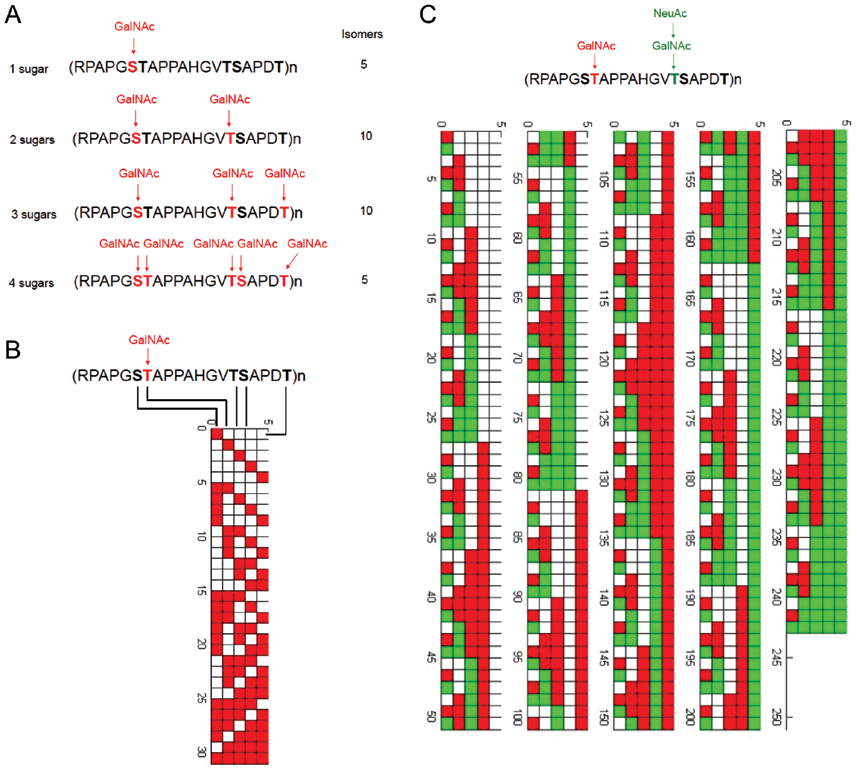
Theoretical glycopeptide epitopes expressed by MUC1. (A) MUC1 protein is heavily glycosylated in the tandem repeat domain of 20 amino acids. Each TR domain contains 5 potential O-glycosylation sites. We have generated a database of glycopeptide sequences with 1, 2, 3, 4, and 5 GalNAc residues, respectively. Of 31 possible MUC1 glycopeptides that vary in number and location of Tn epitopes, four MUC1 sequences that bear one, two, three, or four Tn moieties are illustrated. For TR domain which may bear 1 GalNAc residue, there may exist 5 different isomers for antibody recognition. For TR domain which bears 2 GalNAc residues, there may exist 10 different isomers for antibody recognition. For TR domain which bears 3 GalNAc residues, there may exist 10 different isomers for antibody recognition. For TR domain which bears 4 GalNAc residues, there may exist 5 different isomers for antibody recognition. In reality, cross-reactivity must be considered for monoclonal antibody recognition, thus the exact number of epitopes which may be uniquely recognized by monoclonal antibodies must be determined by experiments. (B) All possible modification results by GalNAc sugar (31 results in total) in a single sequence of RPAPGSTAPPAHGVTSAPDT, as constructed by MATLAB language. (C) All possible modification results (242 results in total) by GalNAc and NeuAc sugars in a single sequence of RPAPGSTAPPAHGVTSAPDT.

We have analyzed the family of mucin glycoproteins, focusing on tandem repeat sequences which are heavily O-glycosylated, and express Tn antigens in cancer conditions. A theoretical database covering possible O-glycopepetide sequences has been constructed by MATLAB language. Fig. 2 shows the example of MUC1 tandem repeat modified by Tn antigen (GalNAc) and sialyl Tn antigen (NeuAcα6GalNAc).

In a MATLAB program specially designed to create Fig. 2B, each of the five modifiable loci was represented by binary digits. After simple calculation, a 5x31 matrix with all zeros was created. Functions existing in MATLAB were utilized to change zeros to ones in the matrix sequentially. Each element of zero represents a locus without modification, while one represents a locus with modification of GalNAc. Finally, every element in the matrix was inputed into a loop, creating a figure where X-axis is five rectangles representing five loci in (RPAPGSTAPPAHGVTSAPDT)n and Y-axis is different modification results (each modified locus was stained by red color).

Based on the same principle, Fig. 2C was created, ternary digits were utilized to represent different modification. Loci with modification of GalNAc only was stained by red color. The combination of GalNAc and NeuAc was stained by green color.

### Biotinylated (glyco)peptides

**Figure 3:**
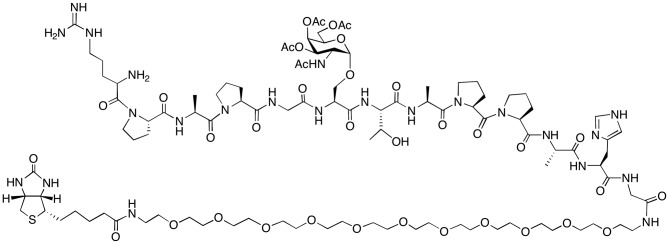
Chemical structure of RPAPGS(Ac_3_GalNAc)TAPPAHG. This synthetic glycopeptide was biotinylated for immobilization on streptavidin-coated plates in ELISA experiments.

The biotinylated glycopeptide RPAPGS(Ac_3_GalNAc)TAPPAHG-dPEG™11-Biotin (Fig. 3), and non-glycosylated peptide, RPAPGTAPPAHG-dPEG™11-Biotin were custom synthesized by Peptide International Inc. (Louisville, KY). They were synthesized on an automated peptide synthesizer from Protein Technologies, Inc. (Tucson, AZ), model ‘Prelude’, using fluorenylmethyloxycarbonyl (Fmoc)-protected amino acids as the building blocks, 6-chloro-benzotriazole-1-yl-oxy-tris-pyrrolidino-phosphonium hexafluorophosphate (PyClock) as the coupling reagent, and 2-chlorotrityl resin preloaded with glycine, loading capacity 0.59 mmol/g, as the solid support. In each coupling cycle PyClock and the Fmoc amino acid were used in 2.5-fold excess, and N-methylmorpholine (NMM) in 4.25-fold excess in N,N-dimethylformamide (DMF). Removal of the Fmoc group after each coupling was performed with 20% piperidine in DMF. For the glycopeptide preparation, the glycosylamino acid Fmoc-Thr(Ac_3_GalNAc)-OH was coupled in only 1.2-fold excess with the coupling reagent O-(7-Azabenzotriazol-1-yl)-N,N,N′,N′-tetramethyluronium hexafluorophosphate (HATU) and NMM. The peptide and glycopeptide were released from the resin with simultaneous deprotection by treatment with cocktail R (TFA/thioanisole/EDT/anisole, 90:5:3:2) and precipitated by cold ether. The crude peptides were purified by preparative reversed-phase HPLC. The peptides were biotinylated via the heterobifunctional cross linker mono-N-t-Boc-amido-dPEG^®^11 amine. Coupling was performed with diphenylphosphoryl azide and NMP in solution. Peptides were characterized by mass spectrometry.

The glycopeptide RPAPGS(GalNAc)TAPPAHG-dPEG™11-Biotin was prepared by deacetylation of RPAPGS(Ac_3_GalNAc) TAPPAHG-dPEG™11-Biotin. Briefly, RPAPGS(Ac_3_GalNAc) TAPPAHG-dPEG™11-Biotin (600 *μ*g in 100 *μ*l DMSO solution) was mixed with 5 *μ*l hydrazine. The mixture was shaken at room temperature for 30 min. The deacetylation reaction was monitored by analytical HPLC on a Waters 626 HPLC instrument with a Symmetry300™ C18 column (5.0 *μ*m, 4.6x250 mm) at 40°C, eluted with a linear gradient of 0–60% MeCN containing 0.1% TFA within 20 min at a flow rate of 1 ml/min. Preparative HPLC was performed on a Waters 600 HPLC instrument with a preparative Symmetry300 C18 column (particle size 7.0 *μ*m, dimensions 19x250 mm), which were eluted with a suitable gradient of aqueous acetonitrile containing 0.1% TFA at a flow rate of 12 ml/min. The residue was neutralized with AcOH until pH 4.0, and subject to the preparative HPLC purification to give the de-acetylated product (420 *μ*g as quantified by analytical HPLC).

The glycopeptide containing the tri-O-acetylated GalNAc residue (synthetic precursor of the de-O-acetylated glycopeptide, RPAPGS(Ac_3_GalNAc)TAPPAHG-dPEG™11-Biotin) was included in the binding studies in order to answer the question whether OH-3, OH-4, and OH-6 of the GalNAc residue were important for the binding specificity of the mAbs. ELISA experiments with the non-glycosylated peptide allows for clues of the extent of peptide involvement in binding.

### Mass spectrometry analysis of (glyco)peptides

Glycosylated and non-glycosylated peptides were diluted in 80% ACN/0.1% FA to a final concentration of 0.1 *μ*g/*μ*l and 1 *μ*l was directly injected at 300 nl/min into an ESI-linear ion trap-mass spectrometer (LTQ XL, Thermo-Fisher Scientific), equipped at the front end with a nano-electrospray ionization source (Thermo-Fisher Scientific). MS spectra were collected in positive-ion mode for 120 sec, at the 400–2,000 m/z range, and the ions of interest were subjected to collision-induced dissociation (35% normalized collision energy).

### Generation of a monoclonal antibody specific for RPAPGS-(GalNAc)TAPPAHG

cDNA containing MUC1 tandem repeat sequences was generated from the breast cancer cell line T47D by a RT-PCR kit from Invitrogen (Carlsbad, CA), with PCR primers 5′-atgacaccgggcacccagtctcct-3′ and 5′-tcaggggagcatggggaaggaaaag-3′. The amplified cDNA sequence was compared to published literature (8, GenBank: X52228.1), and cloned into vector pcDNA-IRES-eGFP (Invitrogen, Carlsbad, CA). The Jurkat cell line, which synthesizes abnormal glycans (O-GalNAc residue) on all glycoproteins (14), was transfected by pcDNA-IRES-eGFP-MUC1 and the mock vector pcDNA-IRES-eGFP, respectively. Stable cell lines were generated by selection with G418, and fluorescence-activated cell sorting for eGFP expressing cells. C57B6 mice were immunized by Jurkat pcDNA-IRES-eGFP-MUC1. Monoclonal antibodies were generated by screening against each synthesized glycopeptide using ELISA methods as described below. One monoclonal antibody, 16A, has been generated, which showed stronger binding to RPAPGS(Ac_3_GalNAc)TAPPAHG in ELISA experiments than the non-glycosylated RPAPGSTAPPAHG. Another monoclonal antibody, 14A, which showed similar binding to RPAPGS(Ac_3_GalNAc)TAPPAHG and RPAPGSTAPPAHG, was also generated.

### ELISA to determine antibody binding to glycopeptides

The biotinylated (glyco)-peptides (1 *μ*g/ml) were bound to streptavidin-coated plates (2 *μ*g/ml), and incubated with serially diluted serum for 2 h. Binding of glycopeptide-specific IgG was visualized by a secondary antibody (goat anti-mouse IgG) followed by colorimetric detection. One percent bovine serum albumin was used as blank for determining the cutoff value.

### Immunohistochemistry

The study subjects were female patients selected from a clinical database at the University of Texas M.D. Anderson Cancer Center. The institutional review boards (IRB) of the M.D. Anderson Cancer Center approved the retrospective review of the medical records and identification and analysis of tumor blocks for the purposes of the present study. Breast cancer diagnosis was made by core needle or excisional biopsy of the breast tumor. All pathologic specimens were reviewed by dedicated breast pathologists. The histological type of the tumor specimens was defined according to the World Health Organization Classification System (15).

Immunohistochemistry was performed as previously described (16). Briefly, 5-*μ*m paraffin-fixed tissue sections were deparaffinized in xylene and rehydrated through using a gradient of alcohol (100, 95 to 80%, Sigma, St. Louis, MO). Antigen retrieval was carried out for 30 min using PT Module (Lab Vision Corp., USA) in Tris-EDTA buffer (pH 9.0). After cooling down, the slides were thoroughly washed in distilled water and washed three times in 1X phosphate-buffered saline (PBS), 2 min each. Endogenous peroxidase activity was quenched by immersion in 3% hydrogen peroxide (Sigma), then in methanol for 10 min at room temperature followed by rinsing for 2 min in 1X PBS three times. Nonspecific binding of the primary antibody was blocked by incubating the sections with 10% normal horse serum for 30 min at room temperature. Sections were then incubated with primary anti-16A, 14A, or C595 (17, Abcam, Cambridge, MA) 4°C overnight, at 1 *μ*g/ml concentration.

The second day, after washing three times in 1X PBS (2 min each), the slides were incubated with secondary anti-mouse IgG-biotin antibody (1:200, Vectastain Elite ABC kit; Vector laboratories, CA, USA) at room temperature for 1 h and rinsed in 1X PBS three times (2 min each). After another 1-h incubation with the avidin-biotin peroxidase complex (1:100, Vectastain Elite ABC Kit; Vector Laboratories, CA, USA) and repeated washing steps with 1X PBS, visualization was performed with the chromagen 3,3′-diaminobenzidine (DAB, Dako, Carpinteria, CA, USA). The slides were counterstained with hematoxylin and coverslipped with PerMount. Sections of Jurkat-pcDNA-IRES-eGFP-MUC1 and Jurkat-pcDNA-IRES-eGFP were used as positive and negative controls, respectively. Isotype IgG and omission of the primary antibody were used as negative controls for staining.

### Surface plasmon resonance (SPR) measurement of antibody affinity toward glycopeptides

Interactions of RPAPGSTAPPAHG, RPAPGS(Ac_3_GalNAc)TAPPAHG, and RPAPGS(GalNAc)TAPPAHG with immobilized antibody 14A and 16A were determined by surface plasmon resonance on a Biacore T100 (GE Healthcare) instrument (18). Antibody 14A and 16A were immobilized on a CM5 chip until reaching 5000 response units. A reference channel was immobilized with ethanolamine, respectively. Immobilizations were carried out at protein concentrations of 25 *μ*g/ml in 10 mM acetate pH 5.0 by using an amine coupling kit supplied by the manufacturer. Measurements were carried out at 25°C in 10 mM HEPES, pH 7.4 containing 150 mM NaCl and 0.005% surfactant P20 at a flow rate of of 30 *μ*l/min. The association time was 120 sec and dissociation time was 200 sec. The surface was regenerated by 3 M MgCl_2_ solution. Data were analyzed with BIA evaluation software (GE Healthcare).

## Results and Discussion

### Generation of a monoclonal antibody, 16A, toward glycopeptide RPAPGS(GalNAc)TAPPAHG

Immunizing mice by MUC-1 transfected mutant cancer cells induced IgG antibody responses against authentic glyco-epitopes expressed on tumor cell surface, which could be measured by ELISA experiments using chemically synthesized glycopeptide RPAPGS(Ac_3_GalNAc)TAPPAHG. We observed individual variation of IgG titers, and selected one mouse with titer above 5000 for hybridoma fusion. Supernatants of hybridoma cultures were screened toward both glycopeptide RPAPGS(Ac_3_GalNAc) TAPPAHG and non-glycosylated peptide RPAPGSTAPPAHG.

**Figure 4:**
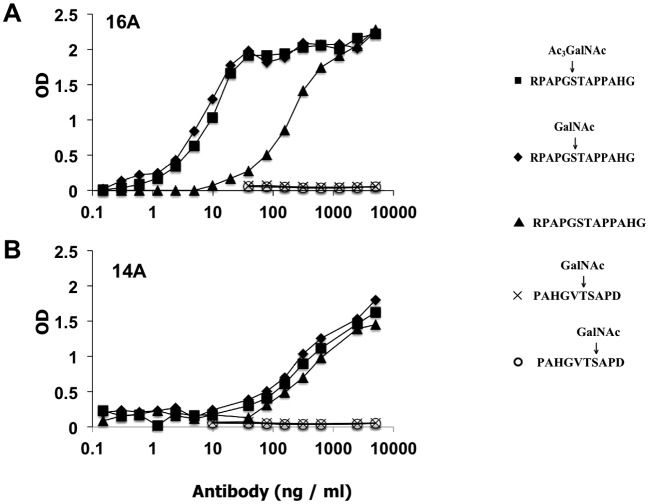
A monoclonal antibody, 16A, binds to glycopeptide RPAPGS(GalNAc) TAPPAHG with high affinity. (A) Monoclonal antibody 16A was prepared as described in the text. Its binding to glycopeptides RPAPGS(GalNAc) TAPPAHG (▪), RPAPGS(Ac_3_GalNAc)TAPPAHG (♦) was compared to peptide control RPAPGSTAPPAHG (▴), 2 *μ*g/ml of biotinylated peptides were bound to streptavidin coated ELISA plates. Monoclonal antibody was added at indicated concentration, and binding was detected by secondary goat anti-mouse IgG antibody, which was conjugated to HRP. At a working concentration of 10 ng/ml, 16A antibody showed strong binding to glycopeptide, but much weaker binding to peptide alone. (B) A control monoclonal antibody, 14A, was generated by screening the supernatant of hybridomas against nonglycosylated control peptide RPAPGSTAPPAHG. 14A antibody showed same binding to glycosylated and non-glycosylated peptides. Both 16A and 14A antibodies showed no reactivity with 2 irrelevant glycopeptides modified by GalNAc, PAHGVT(GalNAc)SAPD and PAHGVTS(GalNAc)APD.

A hybridoma clone 16A, which produces a monoclonal antibody belonging to the IgG1 subclass, showed stronger binding to RPAPGS(Ac_3_GalNAc)TAPPAHG (EC_50_=6.509±0.8019 ng/ml), but approx. 40-fold weaker binding to non-glycosylated peptide, RPAPGSTAPPAHG (EC50=247.3±16.29 ng/ml), as measured by ELISA experiments (Fig. 4A). EC_50_ of binding to RPAPGS(GalNAc)TAPPAHG by 16A was 9.278±1.059 ng/ml, indicating at least 25-fold higher affinity as compared to RPAPGSTAPPAHG.

As a control, we also selected another hybridoma, 14A, by ELISA using RPAPGSTAPPAHG peptide. Not surprisingly, 14A monoclonal antibody showed same binding specificity toward RPAPGS(Ac_3_GalNAc)TAPPAHG and non-glycosylated RPAP GSTAPPAHG. EC_50_ for RPAPGS(Ac_3_GalNAc)TAPPAHG, RPAPGS(GalNAc)TAPPAHG, and RPAPGSTAPPAHG were 189.3±52.55 ng/ml, 245.7±54.80 ng/ml, and 348.3±79.25 ng/ml respectively (Fig. 4).

The 16A and 14A antibodies do not bind to two other GalNAc modified glycopeptides synthesized by our group, PAHGVT(GalNAc)SAPD, or PAHGVTS(GalNAc)APD, as measured by ELISA experiments, indicating that antibody binding is not only toward the GalNAc sugar residue, but also to the specific peptide backbone involved.

However, surface plasmon resonance measurement of the antibody affinity to glyopeptides showed similar binding of 16A antibody toward both glycosylated and non-glycosylated peptides (Table II). This suggests that the 25-fold higher affinity of 16A antibody binding to glycosylated peptides observed in ELISA experiments (Fig. 4) might be due to the conformational changes of glycopeptides when the 2-fragment antigen-binding sites of IgG1 molecules bind to glycopeptides in a bivalent fashion.

### Binding of 16A antibody to patient samples

**Figure 5:**
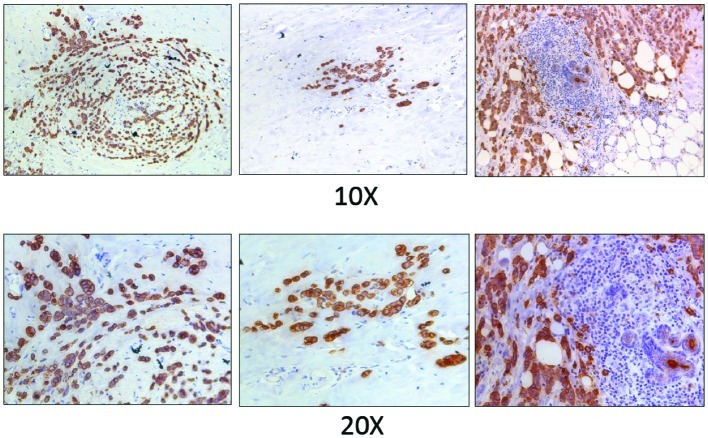
Binding of 16A antibody to tumor samples. Staining of tissue section from a patient with an ER^+^PR^+^HER^+^ breast tumor. Paraffin tissue section was stained by 16A monoclonal antibody at 5 *μ*g/ml, followed by an HRP conjugated secondary antibody. Brown staining represents 16A epitope expression.

We further examined whether 16A antibody binds to breast cancer tissue sections. Among 10 patients studied, we observed strong positive staining in four patients (Table I). Fig. 5 shows a representative staining of an estrogen receptor, progesterone receptor, and HER2-positive breast tumor. In contrast, 14A antibody showed very weak binding in immunohistochemistry experiments (data not shown).

### Problems with current approaches to the discovery of MUC1 biomarkers

In the past, most approaches focused on targeting nonglycosylated MUC1, or MUC1 peptides with undefined glycosylation profiles (19). While the importance of the glycosylation pattern for the immunogenic properties of a glycoprotein has been recognized (20), the peptide portion should not be neglected. Recently, Schietinger and coworkers found that in a mouse fibrosarcoma, a mutant chaperone abolished function of a glycosyltransferase, which disrupted O-glycan core 1 synthesis, and created a transmembrane protein as a tumor-specific antigen. This antigen was recognized by a monoclonal antibody with exquisite specificity. X-ray-crystallographic analysis showed that the cognate epitope consisted of both the Tn antigen and an octapeptide portion of the underlying protein backbone (21). Furthermore, the glycopeptide-specific antibody showed high affinity toward cancer antigen (Kd=10^−7^ M) and cured cancer in mouse models, which is in contrast to the low affinities often observed for antibodies toward Tn antigen (GalNAc). This result suggests that glycopeptide epitopes may be highly immunogenic, most likely more immunogenic than the saccharide portion alone.

Since biochemical characterization of glycan epitopes are often challenging, we have developed a complementary approach, that is to predict the glycolipid or glycopeptide structures based on the central dogma of glycobiology proposed by Kornfeld and Kornfeld (22). Since the glycoconjugates are assembled stepwise by glycosyltransferases, we have written computer programs that predict the glycan structures by sequential additions of sugar units. Here we take a novel approach to the potential discovery of new MUC1 biomarkers via monoclonal antibodies. This approach is based on the immunization of mice with authentic tumor cell surface MUC1. The MUC1 epitopes expressed on the cell surface trigger B cell responses, while the CD4 helper signals are provided by tumor proteins (Fig. 1), when tumor cells are lysed by xenogenicity-induced cell lysis. From these experiments, an entire library of monoclonal antibodies can potentially be obtained. As a first proof-of-principle experiment, we have generated monoclonal antibodies which show higher affinity to glycopeptide *RPAPGS(GalNAc) TAPPAHG*. This approach may be complemented with conventional monoclonal antibody production via immunization of mice with certain MUC1 glycopeptides conjugated to adjuvants (23). Our approach could potentially lead to a massive toolbox of monoclonal antibodies that could be used for the discovery of authentic biomarkers on cancer cell surfaces, and for the development of new tools for diagnostic imaging using radioisotope-labeled monoclonal antibodies.

Cross reactivity is an issue of all monoclonal antibodies. In this study, we generated 16A monoclonal antibody which preferentially binds to glycosylated peptide. The 16A antibody showed strong binding to glycopeptide RPAPGS(GalNAc) TAPPAHG, but much weaker binding to peptide RPAPGSTAPPAHG alone. It has no cross reactivity with 2 other glycopeptides modified by GalNAc, PAHGVT(GalNAc)SAPD and PAHGVTS(GalNAc)APD (Fig. 4). Identifying individual glycopeptide epitopes will need not one, but an entire set of monoclonal antibodies. Thus, multiple monoclonal antibodies with preferential binding specificities will provide specific information for an individual MUC1 glycopeptide.

In conclusion, glycopeptide epitopes expressed by MUC1 may be predicted by bioinformatics tools. Glyco-epitopes expressed on tumor cell surfaces may elicit antibody responses in mice and cancer patients. Monoclonal antibodies can be generated from mice immunized by tumor cells, and selected by ELISA experiments using chemically synthesized glycopeptides predicted by bioinformatics tools. Such monoclonal antibodies are valuable tools for discovery of new biomarkers and targets for immunotherapy. This approach was successful in generating a monoclonal antibody, 16A, that was able to stain tissue sections. The nonglycosylated peptide is being recognized, but 25-fold weaker than the glycopeptide. Interestingly, 16A binds 3,4,6-tri-O-acetylated GalNAc glycopeptide more strongly than the nonglycosylated peptide by a factor of approximately 40, indicating that the absence of hydroxyl groups in the sugar moiety does not abolish binding. One possible explanation for this finding could be that the antibodies engage in intermolecular contacts simultaneously with the peptide and those parts of the Ac_3_GalNAc moiety that it has in common with GalNAc, for example the acetamido group at position 2. Research investigating this molecular recognition phenomenon is currently underway.

## Figures and Tables

**Table I:** Expression of MUC1 in breast cancer patients as measured by 16A monoclonal antibody.

Patient ID	Histology	ER	PR	HER2	MUC1 (16A)
1	IDC+ILC	+	+	−	+
2	IDC	+	−	+	−
3	IDC	+	−	−	−
4	IDC	+	−	+	+
5	IDC	+	+	+	+
6	IDC	−	−	−	−
7	IDC	+	−	−	+
8	IDC	+	−	−	−
9	IDC	+	−	+	−
10	IDC	−	−	−	−

**Table II:** SPR measurement of dissociation constants for the binding of 14A and 16A monoclonal antibodies to glycopeptides.

(Glyco)peptide	Ka (1/Ms)	Kd (1/s)	KD (nM)	Chi^2^ (RU^2^)
16A				
RPAPGS(Ac_3_GalNAc)TAPPAHG	5.721E+4	0.02794	488.5	18.6
RPAPGS(GalNAc)TAPPAHG	3.353E+4	0.03120	930.4	23.1
RPAPGSTAPPAHG	7208	0.003898	540.8	4.95
14A				
RPAPGS(Ac_3_GalNAc)TAPPAHG	1.600E+5	0.07362	460.2	10.3
RPAPGS(GalNAc)TAPPAHG	1.172E+5	0.05141	438.6	19.4
RPAPGSTAPPAHG	1.218E+4	0.002851	234.0	3.35
